# Characterization of HIV Preexposure Prophylaxis Use Behaviors and HIV Incidence Among US Adults in an Integrated Health Care System

**DOI:** 10.1001/jamanetworkopen.2021.22692

**Published:** 2021-08-26

**Authors:** J. Carlo Hojilla, Leo B. Hurley, Julia L. Marcus, Michael J. Silverberg, Jacek Skarbinski, Derek D. Satre, Jonathan E. Volk

**Affiliations:** 1 Division of Research, Kaiser Permanente Northern California, Oakland; 2 Weill Institute for Neurosciences, Department of Psychiatry and Behavioral Sciences, University of California, San Francisco; 3 Department of Population Medicine, Harvard Medical School, Boston, Massachusetts; 4 Harvard Pilgrim Health Care Institute, Boston, Massachusetts; 5 Department of Infectious Diseases, Kaiser Permanente Oakland Medical Center, Oakland, California; 6 Department of Adult and Family Medicine, Kaiser Permanente San Francisco Medical Center, San Francisco, California

## Abstract

**Importance:**

Long-term follow-up is needed to evaluate gaps in HIV preexposure prophylaxis (PrEP) care delivery and to identify individuals at risk for falling out of care.

**Objective:**

To characterize the PrEP continuum of care, including prescription, initiation, discontinuation, and reinitiation, and evaluate incident HIV infections.

**Design, Setting, and Participants:**

This retrospective cohort study used data from the electronic health records (EHR) at Kaiser Permanente Northern California to identify individuals aged 18 years and older who received PrEP care between July 2012 and March 2019. Individuals were followed up from date of linkage (defined as a PrEP referral or PrEP-coded encounter) until March 2019, HIV diagnosis, discontinuation of health plan membership, or death. Data were analyzed from December 2019 to January 2021.

**Exposures:**

Sociodemographic factors included age, sex, race and ethnicity, and neighborhood deprivation index, and clinical characteristics were extracted from the EHR.

**Main Outcomes and Measures:**

The primary outcomes were attrition at each step of the PrEP continuum of care and incident HIV infections.

**Results:**

Among 13 906 individuals linked to PrEP care, the median (interquartile range [IQR]) age was 33 (27-43) years, 6771 individuals (48.7%) were White, and 13 227 (95.1%) were men. Total follow-up was 26 210 person-years (median [IQR], 1.6 [0.7-2.8] years). Of individuals linked to PrEP care, 88.1% (95% CI, 86.1%-89.9%) were prescribed PrEP and of these, 98.2% (95% CI, 97.2%-98.8%) initiated PrEP. After PrEP initiation, 52.2% (95% CI, 48.9%-55.7%) discontinued PrEP at least once during the study period, and 60.2% (95% CI, 52.2%-68.3%) of these individuals subsequently reinitiated. Compared with individuals aged 18 to 25 years, older individuals were more likely to receive a PrEP prescription (eg, age >45 years: hazard ratio [HR], 1.21 [95% CI, 1.14-1.29]) and initiate PrEP (eg, age >45 years: HR, 1.09 [95% CI, 1.02-1.16]) and less likely to discontinue (eg, age >45 years: HR, 0.46 [95% CI, 0.42-0.52]). Compared with White patients, African American and Latinx individuals were less likely to receive a PrEP prescription (African American: HR, 0.74 [95% CI, 0.69-0.81]; Latinx: HR, 0.88 [95% CI, 0.84-0.93]) and initiate PrEP (African American: HR, 0.87 [95% CI, 0.80-0.95]; Latinx: HR, 0.90 [95% CI, 0.86-0.95]) and more likely to discontinue (African American: HR, 1.36 [95% CI, 1.17-1.57]; Latinx: 1.33 [95% CI, 1.22-1.46]). Similarly, women, individuals with lower neighborhood-level socioeconomic status (SES), and persons with a substance use disorder (SUD) were less likely to be prescribed (women: HR, 0.56 [95% CI, 0.50-0.62]; lowest SES: HR, 0.72 [95% CI, 0.68-0.76]; SUD: HR, 0.88 [95% CI, 0.82-0.94]) and initiate PrEP (women: HR, 0.71 [95% CI, 0.64-0.80]; lower SES: HR, 0.93 [95% CI, 0.87-.0.99]; SUD: HR, 0.88 [95% CI, 0.81-0.95]) and more likely to discontinue (women: HR, 1.99 [95% CI, 1.67-2.38]); lower SES: HR, 1.40 [95% CI, 1.26-1.57]; SUD: HR, 1.23 [95% CI, 1.09-1.39]). HIV incidence was highest among individuals who discontinued PrEP and did not reinitiate PrEP (1.28 [95% CI, 0.93-1.76] infections per 100 person-years).

**Conclusions and Relevance:**

These findings suggest that gaps in the PrEP care continuum were concentrated in populations disproportionately impacted by HIV, including African American individuals, Latinx individuals, young adults (aged 18-25 years), and individuals with SUD. Comprehensive strategies to improve PrEP continuum outcomes are needed to maximize PrEP impact and equity.

## Key Points

**Question:**

What factors are associated with gaps in the HIV preexposure prophylaxis (PrEP) continuum of care and where in the continuum do HIV infections occur?

**Findings:**

In this cohort of 13 906 insured adults linked to PrEP care, individuals aged 18 to 25, African American individuals, Latinx individuals, women, individuals with lower socioeconomic status, and individuals with a substance use disorder were more likely to experience gaps in the PrEP continuum of care. Attrition along the continuum was associated with incident HIV infection.

**Meaning:**

These findings suggest that comprehensive strategies are warranted to improve PrEP continuum of care outcomes in high-priority populations.

## Teaser

This cohort study assesses factors associated with receipt and use of preexposure prophylaxis and incidence of HIV among individuals assessed for preexposure prophylaxis.

## Introduction

While the overall number of new HIV diagnoses in the US has declined in recent years, African American individuals, Latinx individuals, young adults, men who have sex with men, and individuals with alcohol and other substance use disorders (SUD) continue to experience a disproportionate burden of new HIV infections.^1,2^ HIV preexposure prophylaxis (PrEP) is highly effective in reducing the risk of acquiring HIV^3,4^ and is a crucial component of the Ending the HIV Epidemic Initiative,^5^ an ambitious plan with the goal of reducing HIV incidence in the US by 90% within 10 years.

The PrEP continuum of care provides a framework for measuring progress toward national HIV prevention goals and for evaluating factors associated with optimal PrEP delivery. The continuum involves a series of steps: linkage to PrEP care, prescription of medication, initiation of therapy, persistence on PrEP throughout periods of risk, and reinitiation of PrEP among individuals who discontinue.^6,7^ In our 2017 study,^8^ we found that falling out of care along these steps was associated with incident HIV infections. Thus, characterizing gaps at each step of the PrEP continuum of care and identifying individuals at risk of attrition could facilitate the development and prioritization of interventions to maximize PrEP impact and equity. Although several studies have evaluated the PrEP continuum in real-world settings, existing data have largely relied on small patient samples or administrative insurance databases with incomplete clinical information.^9,10,11,12,13,14,15^ Comprehensive long-term follow-up data are needed to accurately evaluate PrEP continuum outcomes and identify individuals most at risk for falling out of care and acquiring HIV.

In this study, we examined the PrEP continuum of care in a large cohort of individuals linked to PrEP services at Kaiser Permanente Northern California (KPNC). The primary objectives of the study were to identify gaps in the PrEP continuum of care, evaluate demographic and clinical factors associated with attrition, and characterize HIV infections at each step of the PrEP continuum.

## Methods

### Study Design and Data Collection

This retrospective cohort study was approved by the KPNC institutional review board with a waiver of informed consent because the study used existing clinically derived data and involved no more than minimal risk. This study followed the Strengthening the Reporting of Observational Studies in Epidemiology (STROBE) reporting guideline.

KPNC is a large health care system that provides comprehensive care, including integrated pharmacy and laboratory services, to 36% of the insured population in California.^16^ The PrEP program at KPNC has been described previously.^17,18^ We used data from the electronic health record (EHR) to systematically identify patients aged 18 years or older linked to PrEP care between July 16, 2012 (ie, the date of PrEP regulatory approval in US^19^), and March 31, 2019. Linkage was defined as having a PrEP referral or a PrEP-coded clinical encounter. At KPNC, primary care practitioners and other clinicians refer patients to specialty PrEP clinicians for further evaluation and management.^18^ KPNC has a database that indexes referrals and uses a unique code for PrEP-related encounters. PrEP-coded clinical encounters are sensitive markers for PrEP linkage, with 95.9% of individuals using PrEP having a PrEP-coded encounter. Individuals were included in our analysis if they were KPNC health plan members for at least 6 months during the study period and had active insurance coverage at the time of PrEP linkage and initiation. Individuals were followed from the date of PrEP linkage until the end of the study, HIV diagnosis, discontinuation of health plan membership (defined as a loss in KPNC membership for ≥3 months), or death.

Sociodemographic characteristics at time of PrEP linkage, including age, self-reported race and ethnicity, sex, and insurance type (ie, public or commercial) were extracted from administrative databases. Race and ethnicity were examined because they have been identified in the literature as factors associated with access to health care and risk of HIV infection. The Neighborhood Deprivation Index (NDI), a marker of neighborhood-level socioeconomic status (SES), was measured for each participant using their zip code at the time of PrEP linkage.^20^ Bacterial sexually transmitted infections (STIs; includes syphilis, gonorrhea, and chlamydia), pharmacy records, and alcohol use disorder and SUD diagnoses were extracted from the EHR (eTable in the Supplement). We evaluated alcohol and SUD as clinical risk factors, as these are well-established risk factors of poor adherence and care engagement in the PrEP and HIV treatment literature.^21,22,23^ Insurance type and NDI were missing in less than 0.5% of all participants and were handled using listwise deletion. Incident HIV diagnoses were identified using a KPNC HIV registry.^24^ EHR review was independently performed by 2 of us (J.C.H. and J.E.V.) for individuals with incident HIV infections and active PrEP prescriptions to assess self-reported PrEP use prior to seroconversion.

### Outcomes

We evaluated PrEP prescription, initiation, discontinuation, and reinitiation among individuals linked to PrEP care. Prescription was defined as an order for emtricitabine and tenofovir disoproxil fumarate or emtricitabine and tenofovir alafenamide with an indication for PrEP. Initiation was defined as a pharmacy fill, and discontinuation was defined as longer than 120 days without PrEP on hand based on pharmacy refill records.^18^ Individuals who discontinued but had a subsequent pharmacy fill for PrEP were considered to have reinitiated PrEP. Additionally, we identified new HIV diagnoses and evaluated where in the continuum these infections occurred.

### Statistical Analysis

The cumulative proportion of linked individuals who received a PrEP prescription, initiated PrEP, discontinued PrEP, and reinitiated PrEP after discontinuation were estimated using a Kaplan-Meier estimator. Cox models estimated unadjusted hazard ratios (HRs) to evaluate the associations between demographic and clinical characteristics and attrition at each step. For these analyses, alcohol use disorder and SUD diagnoses were treated as time-fixed variables. Follow-up time was separated into distinct intervals for each step of the continuum, and only participants who completed the previous step were included. For example, to estimate HRs for PrEP initiation, analysis was limited to only individuals who received a PrEP prescription with follow-up commencing at the date of PrEP prescription and ending at the date when the prescription was filled (ie, PrEP initiation) or, for those who did not initiate PrEP, when study follow-up ended. For individuals who discontinued and reinitiated PrEP more than once, we only considered the first reinitiation event in our analysis. Tests of linear trend for NDI quintiles were estimated using orthogonal polynomial contrasts.

For a subset of individuals, PrEP was prescribed prior to being linked to care (ie, before referral or PrEP-coded encounter); this included individuals who had been using PrEP and transferred their care to KPNC or individuals whose initial PrEP encounter was not properly coded. For those individuals, we considered their date of PrEP linkage as the date they were first prescribed PrEP in KPNC, and follow-up time in the Cox model was coded as 0 + ε, in which ε is less than the first observed event time (ie, 0.01).^25^ Individuals who discontinued KPNC health plan membership after PrEP initiation were censored at the last date of membership. Schoenfeld residuals and log − log plots were assessed to test the proportional hazards assumption.

We calculated the HIV incidence rate overall and at each step of the PrEP continuum. Associated 95% CIs were estimated based on a Poisson distribution. All analyses were conducted using Stata statistical software version 14.2 (StataCorp). *P* values were 2-sided, and statistical significance was set at *P* = .05. Data were analyzed from December 2019 to January 2021.

## Results

### Patient Characteristics

The total analytical sample included 13 906 patients (median [interquartile range] age, 33 [27-43] years; 13 227 [95.1%] men) (Table 1). Among the sample, 6671 individuals (48.7%) were White, 2997 individuals (21.6%) were Latinx, and 971 individuals (7.0%) were African American. A total of 542 individuals (3.9%) had public health insurance. Additionally, 2255 individuals (16.2%) were diagnosed with a bacterial STI in the year prior or within 30 days of PrEP linkage. Patients were followed for a total of 26 210 person-years after PrEP linkage (median [interquartile range], 1.6 [0.7-2.8] years). A study cohort flowchart, including reasons for censoring, is presented in the eFigure in the Supplement.

**Table 1.** Demographic and Clinical Characteristics of Patients Linked to PrEP Care at Kaiser Permanente Northern California from July 2012 to March 2019

Characteristic	No. (%) (N = 13 906)
Age, y	
18-25	2720 (19.6)
26-35	5350 (39.8)
36-45	2910 (20.9)
>45	2746 (19.8)
Race and ethnicity	
White	6771 (48.7)
Latinx	2997 (21.6)
Asian	2057 (14.8)
African American	971 (7.0)
Other or unknown^a^	1110 (8.0)
Sex recorded in health record	
Male	13227 (95.1)
Female	679 (4.9)
Neighborhood deprivation index, quintile	
First (highest SES)	2779 (20.0)
Second	2774 (20.0)
Third	2854 (20.6)
Fourth	2709 (19.5)
Fifth (lowest SES)	2762 (19.9)
Public health insurance (Medicaid)	542 (3.9)
Alcohol use disorder	3506 (25.2)
Substance use disorder	1090 (7.8)
Bacterial STI in year prior to or within 30 d of PrEP linkage	2255 (16.2)

Abbreviations: PrEP, preexposure prophylaxis; SES, socioeconomic status; STI, sexually transmitted infection (includes gonorrhea, chlamydia, and syphilis).

^a^ Includes individuals reporting more than 1 race or ethnicity.

### PrEP Continuum of Care

The PrEP continuum of care is shown in the Figure. Of 13 906 patients linked to PrEP care, the cumulative proportion of patients prescribed PrEP at the end of the study was 88.1% (95% CI, 86.1%-89.9%). Of those prescribed PrEP, 98.2% (95% CI, 97.2%-98.8%) initiated PrEP, and of those who initiated PrEP, 52.2% (95% CI, 48.9%-55.7%) discontinued at least once during the study period. We observed the highest rates of discontinuation within the first 2 years of starting PrEP. At 2 years, the cumulative proportion of discontinuation was 38.4% (95% CI, 37.2%-39.6%) of individuals. Of those who discontinued PrEP at least once during the study period, 60.2% (95% CI, 52.2%-68.3%) subsequently reinitiated the regimen before the end of follow-up.

**Figure.**
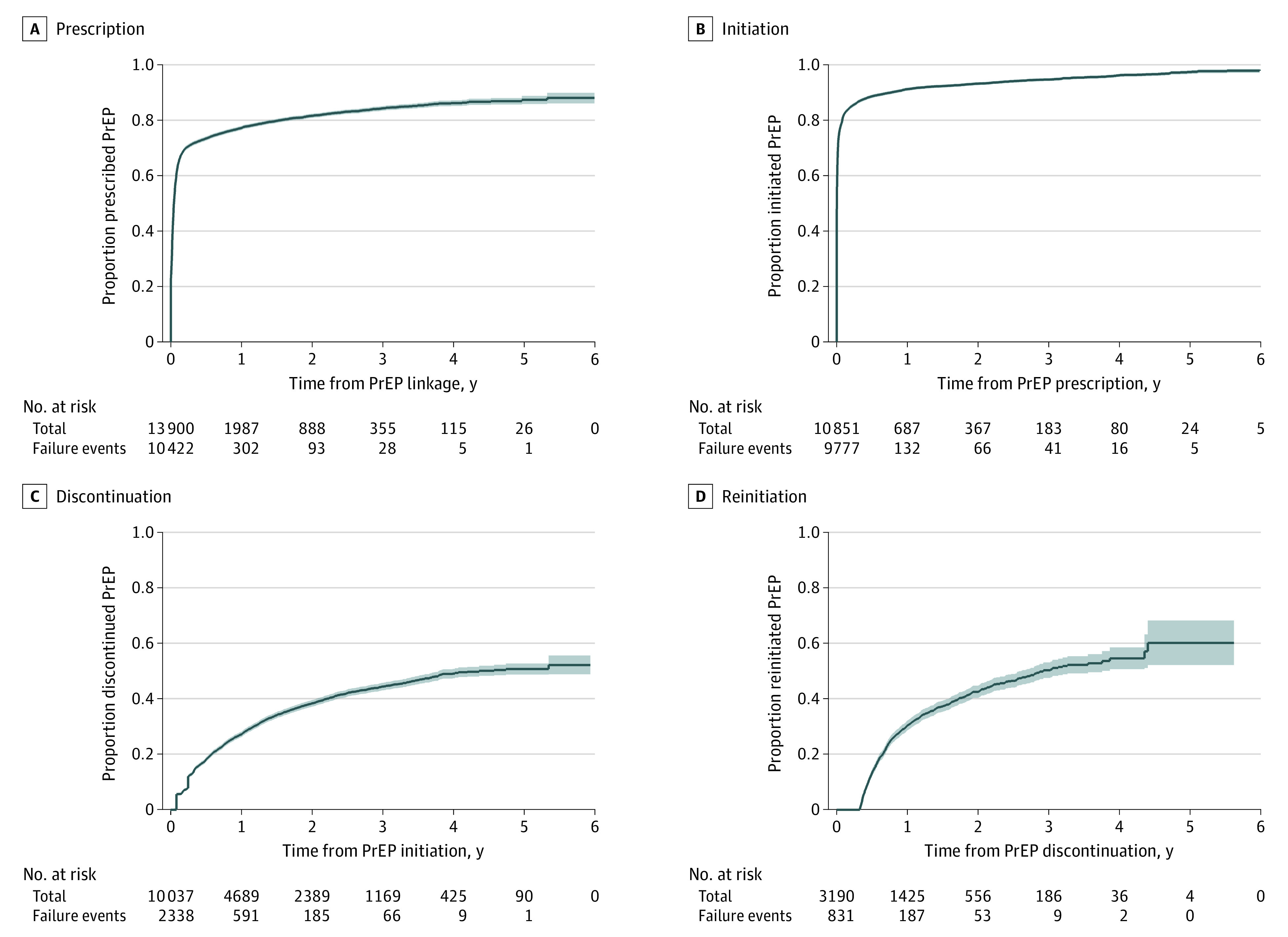
Proportion of Patients Linked to Preexposure Prophylaxis (PrEP) Services at Kaiser Permanente Northern California Who Were Prescribed, Initiated, Discontinued, or Reinitiated PrEP Between July 2012 to March 2019

Table 2 summarizes the demographic and clinical characteristics associated with attrition at each step of the PrEP continuum. Compared with individuals aged 18 to 25 years, individuals in older age groups had higher rates of PrEP prescription (eg, age >45 years: hazard ratio [HR], 1.21 [95% CI, 1.14-1.29]) and initiation (eg, age >45 years: HR, 1.09 [95% CI, 1.02-1.16]), and lower rates of discontinuation (eg, age >45 years: HR, 0.46 [95% CI, 0.42-0.52]). Compared with White individuals, African American individuals were less likely to receive a PrEP prescription (HR, 0.74 [95% CI, 0.69-0.81]) and initiate PrEP (HR, 0.87 [95% CI, 0.80-0.95]) and more likely to discontinue PrEP after initiation (HR, 1.36 [95% 1.17-1.57]). Latinx individuals similarly experienced lower rates of PrEP prescription (HR, 0.88 [95% CI, 0.84-0.93]) and initiation (HR, 0.90 [95% CI, 0.86-0.95]) and higher rates of PrEP discontinuation (HR, 1.33 [95% CI, 1.22-1.46]) compared with White individuals. Among individuals who discontinued PrEP, African American and Asian individuals were more likely to reinitiate PrEP compared with their White counterparts (Table 2). Compared with men, women had lower rates of PrEP prescription (HR, 0.56 [95% CI, 0.50-0.62]) and initiation (HR, 0.71 [95% CI, 0.64-0.80]). Women were also more likely to discontinue PrEP (HR, 1.99 [95% CI, 1.67-2.38]) and less likely to reinitiate PrEP after discontinuation (HR, 0.52 [95% CI, 0.36-0.77]). Individuals with greater neighborhood-level deprivation had lower rates of PrEP prescription (compared with the first [highest SES] quintile: second quintile: HR, 0.90 [95% CI, 0.85-0.95]; third quintile: HR, 0.85 [95% CI, 0.80-0.90; fourth quintile: HR, 0.86 [95% CI, 0.81-0.91]; fifth quintile: HR, 0.72 [95% CI, 0.68-0.76]; linear trend: *P* < .001) and initiation (compared with the first [highest SES] quintile: second quintile: HR, 1.01 [95% CI, 0.95-1.07]; third quintile: HR, 0.96 [95% CI, 0.91-1.02]; fourth quintile: HR, 0.97 [95% CI, 0.91-1.03]; fifth quintile: HR, 0.93 [95% CI, 0.87-0.99]; linear trend: *P* = .008) and higher rates of discontinuation (compared with the first [highest SES] quintile: second quintile: HR, 1.13 [95% CI, 1.01-1.26]; third quintile: HR, 1.21 [95% CI, 1.08-1.35]; fourth quintile: HR, 1.28 [95% CI, 1.15-1.43]; fifth quintile: HR, 1.40 [95% CI, 1.26-1.57]; linear trend: *P* < .001). We also found that SUD was negatively associated with PrEP prescription (HR, 0.88 [95% CI, 0.82-0.94]) and initiation (HR, 0.88 [95% CI, 0.81-0.95]). Of individuals who initiated PrEP, individuals with SUD had a higher rate of discontinuation than those without SUD (HR, 1.23 [95% CI, 1.09-1.39]).

**Table 2.** Demographic and Clinical Factors Associated With Each Step of the PrEP Continuum of Care

Characteristic	PrEP status, hazard ratio (95% CI)			
	Prescription	Initiation	Discontinuation	Reinitiation
Age, y				
18-25	1 [Reference]	1 [Reference]	1 [Reference]	1 [Reference]
26-35	1.33 (1.26-1.41)	1.04 (0.98-1.10)	0.63 (0.58-0.69)	1.10 (0.94-1.30)
36-45	1.41 (1.33-1.50)	1.10 (1.03-1.17)	0.52 (0.46-0.58)	1.06 (0.89-1.28)
>45	1.21 (1.14-1.29)	1.09 (1.02-1.16)	0.46 (0.42-0.52)	0.95 (0.78-1.16)
Race and ethnicity				
White	1 [Reference]	1 [Reference]	1 [Reference]	1 [Reference]
Latinx	0.88 (0.84-0.93)	0.90 (0.86-0.95)	1.33 (1.22-1.46)	1.12 (0.96-1.31)
Asian	0.96 (0.91-1.02)	1.06 (1.00-1.12)	1.06 (0.96-1.18)	1.25 (1.05-1.49)
African American	0.74 (0.69-0.81)	0.87 (0.80-0.95)	1.36 (1.17-1.57)	1.32 (1.04-1.67)
Other or unknown^a^	0.91 (0.84-0.97)	1.03 (0.95-1.11)	1.19 (1.04-1.36)	0.99 (0.78-1.25)
Women	0.56 (0.50-0.62)	0.71 (0.64-0.80)	1.99 (1.67-2.38)	0.52 (0.36-0.77)
Neighborhood Deprivation Index, quintile				
First (highest SES)	1 [Reference]	1 [Reference]	1 [Reference]	1 [Reference]
Second	0.90 (0.85-0.95)	1.01 (0.95-1.07)	1.13 (1.01-1.26)	1.03 (0.86-1.24)
Third	0.85 (0.80-0.90)	0.96 (0.91-1.02)	1.21 (1.08-1.35)	0.93 (0.77-1.12)
Fourth	0.86 (0.81-0.91)	0.97 (0.91-1.03)	1.28 (1.15-1.43)	0.89 (0.74-1.08)
Fifth (lowest SES)	0.72 (0.68-0.76)	0.93 (0.87-0.99)	1.40 (1.26-1.57)	1.04 (0.87-1.26)
Public health insurance	0.78 (0.70-0.87)	0.96 (0.86-1.07)	1.48 (1.24-1.76)	0.89 (0.65-1.22)
Alcohol use disorder	1.08 (1.03-1.12)	1.00 (0.95-1.04)	0.97 (0.90-1.05)	1.14 (1.00-1.30)
Substance use disorder	0.88 (0.82-0.94)	0.88 (0.81-0.95)	1.23 (1.09-1.39)	1.13 (0.92-1.38)
Bacterial STI in year prior to or within 30 d of PrEP linkage	1.17 (1.11-1.23)	0.95 (0.91-1.01)	1.00 (0.91-1.09)	1.21 (1.04-1.41)

Abbreviations: PrEP, preexposure prophylaxis; SES, socioeconomic status; STI, sexually transmitted infection (includes gonorrhea, chlamydia, and syphilis).

^a^ Includes individuals reporting more than 1 race or ethnicity.

### HIV Incidence

In total, 136 individuals (0.98%) were diagnosed with HIV during the study period, of whom 45 (33.1%) were diagnosed during assessment for PrEP eligibility at the time of linkage. Excluding patients who were diagnosed at linkage, the overall HIV incidence rate was 0.35 (95% CI, 0.28-0.43) new infections per 100 person-years, 0.87 (95% CI, 0.63-1.21) new infections per 100 person-years among those who were not prescribed PrEP, 1.06 (95% CI, 0.62-1.83) new infections per 100 person-years among those who were prescribed PrEP but did not initiate the regimen, and 1.28 (95% CI, 0.93-1.76) new infections per 100 person-years among those who discontinued and did not reinitiate PrEP (Table 3). We identified 5 individuals who seroconverted and had a supply of PrEP based on pharmacy records; all 5 self-reported PrEP discontinuation prior to seroconversion. Of those who remained persistent on PrEP, we found no new infections over 9139 person-years of follow-up.

**Table 3.** HIV Incidence Rate Estimates

PrEP status	HIV infections, No./total No. of individuals	Total follow-up, person-years	Incidence (95% CI), per 100 person-years
Overall^a^	91/13 861	26 210	0.35 (0.28-0.43)
Linked but not prescribed PrEP	36/3013	4119	0.87 (0.63-1.21)
Prescribed PrEP but did not initiate	13/811	1226	1.06 (0.62-1.83)
Discontinued but reinitiated PrEP	4/1082	1420	0.28 (0.11-0.75)
Discontinued and did not reinitiate PrEP	38/2108	2973	1.28 (0.93-1.76)
Persistent on PrEP^b^	0/5367	9139	0.00 (0.00-0.04)^c^

Abbreviation: PrEP, preexposure prophylaxis.

^a^ Excludes individuals who were diagnosed with HIV during the eligibility assessment at PrEP linkage.

^b^ Persistent on PrEP refers to individuals who initiated and remained on PrEP throughout follow-up.

^c^ One-sided 97.5% upper CI.

## Discussion

In this cohort study involving more than 13 900 individuals and more than 26 000 person-years of clinical follow-up, overall PrEP uptake was high, as almost 90% of individuals linked to PrEP care received a prescription for PrEP and more than 90% of these initiated the regimen. However, discontinuations were common, particularly within the first 2 years, although nearly two-thirds of those who discontinued later restarted the regimen. Attrition at each step of the PrEP continuum was concentrated in populations disproportionately affected by HIV, including African American individuals, Latinx individuals, young adults (aged 18-25 years), and individuals with SUD.^1,2,26^ Women and individuals with lower SES were also at increased risk of falling out of care at each step of the PrEP continuum, highlighting disparities that may limit the impact of this critical intervention. The incident HIV diagnoses we observed underscore the important public health consequences of gaps in PrEP care delivery and how these lapses can perpetuate existing inequities.

While PrEP uptake in the US has increased steadily since 2012,^27,28^ less than 10% of individuals with an indication for PrEP were prescribed PrEP, with lower uptake among African American and Latinx individuals.^1,29^ In our analysis of an insured population with access to care, we found similar disparities, as African American and Latinx individuals were less likely to receive a PrEP prescription and initiate PrEP compared with White individuals, even after being linked to PrEP care. Other studies have noted low rates of PrEP initiation among members of racial and ethnic minority groups, such as African American individuals.^6,27,30^ For example, in a cohort of young African American men who have sex with men, only 44% of participants not previously using PrEP opted to start despite access to PrEP navigation services.^14^ Reasons why individuals were not prescribed PrEP or why they declined to start PrEP likely reflect multiple cooccurring individual, social, and structural barriers.^31^ In a previous analysis, more than one-third of KPNC survey respondents with recently acquired HIV cited cost as a barrier to PrEP use.^32^ Cost has been widely described as a barrier to PrEP uptake^31,33^ and may partly account for why individuals in our study with lower neighborhood-level SES were less likely to receive a prescription and initiate PrEP despite health insurance coverage. At KPNC, the cost of PrEP has varied substantially among individuals and throughout the study period and has been largely dependent on the type of health plan individuals enroll into. The US Preventive Services Task Force recommendations to provide PrEP at no out-of-pocket cost may help remedy some cost-related barriers for individuals.^34^ Low perceived need, medical mistrust, stigma, clinician apprehension, and structural racism have also been identified as barriers to PrEP prescription and initiation among minoritized groups, including women and individuals with SUD.^31,35,36,37,38,39,40^ Young adults, a demographic group with one of the highest rates of incident HIV,^1,26^ face additional challenges, including limited knowledge and experience navigating health care and reliance on their parents for health insurance coverage.^41,42^

Maximizing the population impact of PrEP is contingent not only on uptake and equity, but also on the extent to which individuals remain on PrEP.^43^ Previous studies, such as a 2020 study by Rutstein et al,^44^ have documented high rates of discontinuation within the first 3 to 6 months of PrEP initiation. However, many individuals may need ongoing support beyond the first several months of PrEP use to ensure sustained use throughout periods of HIV risk.^12,13,45^ An analysis of administrative pharmacy records by Coy et al^13^ found that nearly two-thirds of all PrEP users at a national chain pharmacy had discontinued PrEP by the second year. Our results are consistent with those findings. We found that rates of discontinuation were highest in the first 2 years of PrEP use. For some patients, PrEP discontinuation may reflect a decrease in HIV risk and a deliberate decision that this prevention strategy is no longer needed. However, incident HIV cases observed among those who discontinued PrEP and did not reinitiate and the higher rates of discontinuation in key subgroups disproportionately affected by HIV suggest broader systemic barriers. A study by Huang et al^45^ similarly found that African American individuals, young adults, and women were more likely to stop PrEP after only a brief period of use. Notably, we found that although African American individuals were more likely to discontinue PrEP, they were also more likely to reinitiate PrEP. It is possible that this finding reflects more frequent missed doses because of adherence challenges and longer delays in requesting refills rather than true discontinuation. Regardless, these results suggest interest among many in this group in continuing PrEP, and strategies are needed to mitigate barriers to persistence.

As in other studies,^46,47,48^ overall HIV incidence in our cohort was low. However, a large proportion of cases were diagnosed around the time of PrEP linkage, highlighting the critical need for earlier identification of individuals who are at increased risk for HIV. Leveraging EHR data to estimate risk and develop clinical decision support tools to facilitate PrEP discussions is a promising strategy to detect and prevent HIV infections that occur before linkage to PrEP care.^49,50^ Seroconversions among individuals who did not have HIV at linkage but were not prescribed PrEP and among those who did not initiate PrEP after receipt of a prescription underscore the importance of mitigating barriers to uptake. High rates of HIV among individuals who discontinued and did not reinitiate PrEP emphasize the ongoing need for strategies to support sustained PrEP use during periods of HIV risk and re-engage individuals who have fallen out of care.

### Limitations

Our study has limitations. We relied on pharmacy records and did not directly assess adherence. However, the use of pharmacy refill data has been validated to measure adherence and estimate treatment response among persons living with HIV.^51,52,53^ It is possible that we overestimated discontinuations if individuals were using event-driven PrEP or if there were substantial periods with suboptimal adherence that delayed the need for medication refills. Notably, the use of event-driven PrEP was not formally implemented at KPNC until after the end of the study period,^54^ so the number of individuals using this dosing strategy is likely minimal. We were unable to ascertain reasons for why individuals were not prescribed PrEP or why individuals opted to not start the regimen. HIV incidence may also have been underestimated as a result of loss to follow-up, especially if individuals who discontinued KPNC membership were at higher risk for HIV. Additionally, our study involved an insured cohort comprised predominantly of men, and we did not have data on gender identity. Although KPNC members represent more than one-third of all insured people in California and have demographic characteristics similar to those of non-KPNC members,^16^ the generalizability of our findings may be limited among uninsured, female, and transgender persons.

## Conclusions

This cohort study extends the existing literature by characterizing the PrEP continuum of care in one of the largest cohorts to our knowledge to date, with more than 26 000 person-years of follow-up. A key strength of our study was the use of robust EHR data from an integrated health care system that is the largest health care organization in California.^55^ Encouragingly, we found that rates of PrEP prescription and initiation were high. However, priority populations for PrEP delivery, including members of racial and ethnic minority groups, young adults, women, individuals with lower SES, and individuals with SUD, were less likely to receive a PrEP prescription and initiate PrEP and more likely to discontinue PrEP despite comparable health care access. These findings suggest that health care access alone is not sufficient to optimize PrEP delivery and achieve national HIV prevention goals, including population impact and equity. Comprehensive strategies tailored toward high-priority populations are needed to mitigate attrition along the PrEP continuum of care.

## Notes

Supplement. **eFigure.** Study Cohort Flowchart **eTable.** Definition of Clinical Variables
