# Supplementary material for: Characterization of HIV Preexposure Prophylaxis Use Behaviors and HIV Incidence Among US Adults in an Integrated Health Care System
Source: JAMA Netw Open. 2021 Aug 26;4(8):e2122692. doi: 10.1001/jamanetworkopen.2021.22692 (PMC8391097; doi:10.1001/jamanetworkopen.2021.22692)
Supplement: Supplement. — eFigure. Study Cohort Flowchart eTable. Definition of Clinical Variables [file jamanetwopen-e2122692-s001.pdf]

# Supplemental Online Content

Hojilla JC, Hurley LB, Marcus JL, et al. Characterization of HIV preexposure prophylaxis use behaviors and HIV incidence among US adults in an integrated health care system. *JAMA Netw Open.* 2021;4(8):e2122692. doi:10.1001/jamanetworkopen.2021.22692

**eFigure.** Study Cohort Flowchart
**eTable.** Definition of Clinical Variables

This supplemental material has been provided by the authors to give readers additional information about their work.

© 2021 Hojilla JC et al. *JAMA Network Open.*

**eFigure.** Study Cohort Flowchart

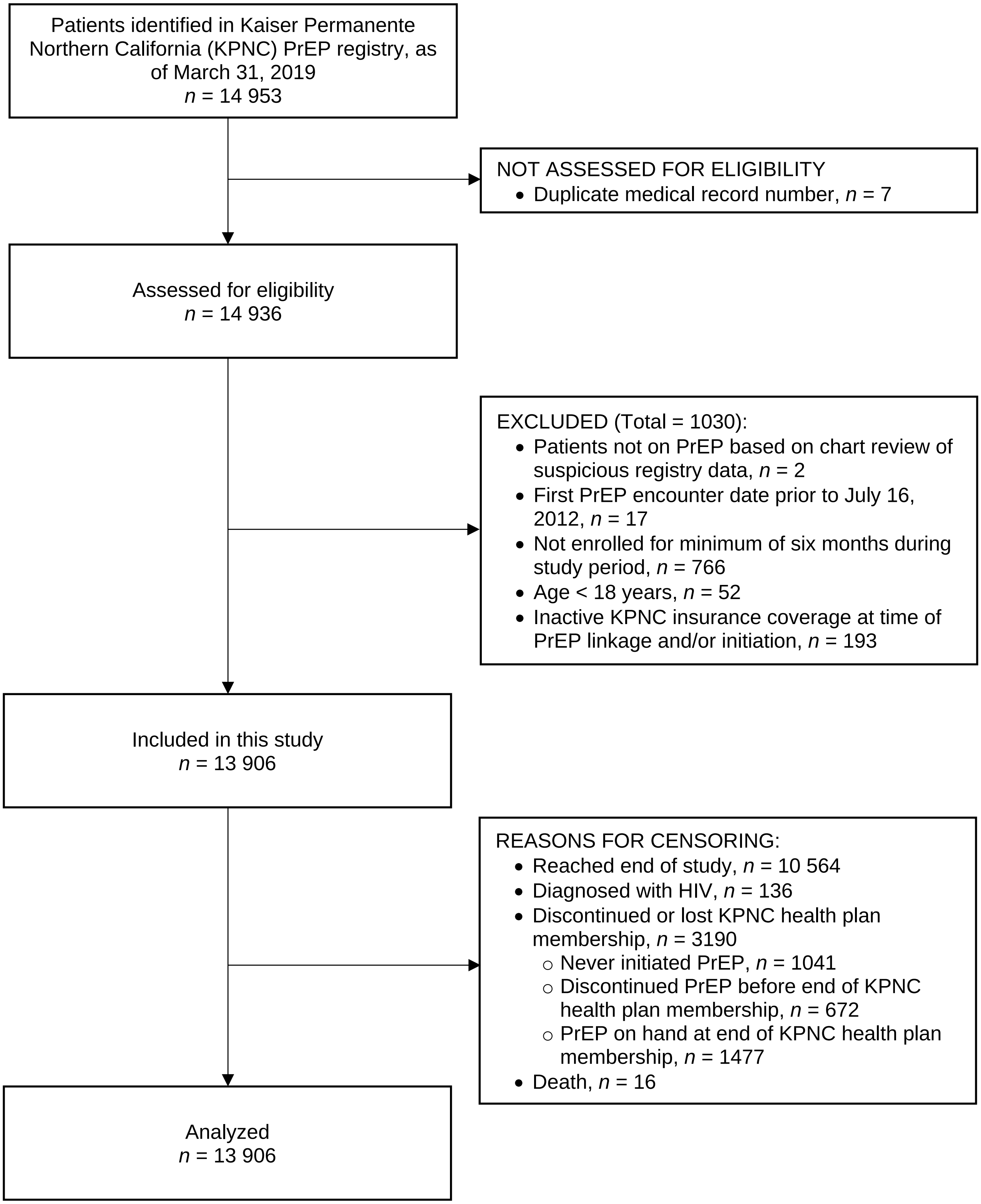


© 2021 Hojilla JC et al. *JAMA Network Open.*

**eTable 1.** Definition of Clinical Variables

| | |
|---|---|
| | **Definition** |
| **Clinical diagnoses** | |
| Alcohol use disorder | ICD-9 291x, 303x<br>ICD-10: F10x |
| Substance use disorder | ICD-9: 292x, 304x, 305x (excluding 305.1)<br>ICD-10: F11x – F19x (excluding F17x), F55x. |
| **Laboratory tests and results** | |
| Incident chlamydia | Positive urethral, rectal, pharyngeal, and/or vaginal/cervicovaginal chlamydia |
| Incident gonorrhea | Positive urethral, rectal, pharyngeal, and/or vaginal/cervicovaginal gonorrhea |
| Incident syphilis | Reactive rapid plasma reagin or positive treponemal IgG and co-incident treatment with benzathine penicillin by intramuscular injection |

ICD = International Classification of Diseases.
Clinical diagnoses and laboratory results documented during the study period, July 16, 2012, to March 31, 2019.

© 2021 Hojilla JC et al. *JAMA Network Open.*
